# New and Unforeseen Crystal Growth Processes for a Metal Oxide

**DOI:** 10.1021/acsomega.3c07772

**Published:** 2023-12-12

**Authors:** Michaela E. Whitehurst, Simon R. Hall

**Affiliations:** School of Chemistry, University of Bristol, Cantock’s Close, Bristol BS8 1TS, U.K.

## Abstract

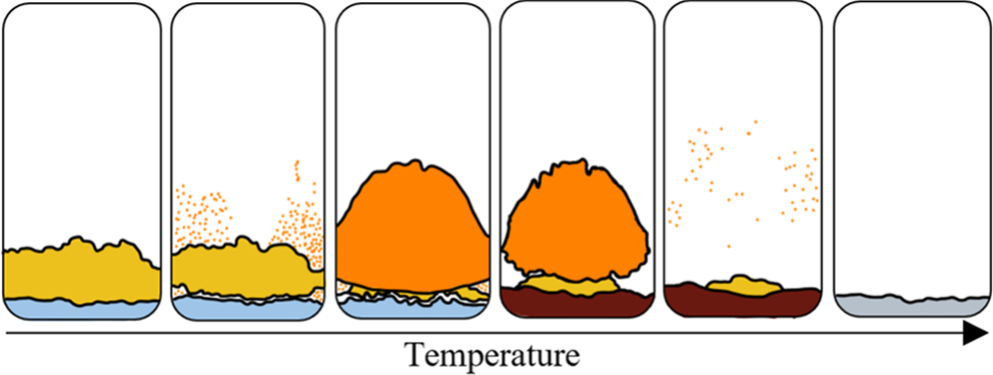

The synthesis of corundum (α-Al_2_O_3_) via a layered Al_2_O_3_–MoO_3_ system was directly observed for the first time. This revealed a new crystal growth process with three key features: (1) the formation of an Al_2_(MoO_4_)_3_ intermediate layer through a solid–solid interaction in the temperature range of ∼705–860 °C; (2) the melting of the Al_2_(MoO_4_)_3_ layer between approximately 870 and 890 °C; and (3) the decomposition of Al_2_(MoO_4_)_3_ to corundum between 950 and 1100 °C. This molten intermediate decomposition (MIND) mechanism produced corundum, which was light bluish-gray in color and was defined in CIE (L* a* b*) color space as L* = 76.65, a* = −1.09, and b* = −6.20. The reagents used in this study were the same as those used in MoO_3_ flux growth studies on the synthesis of corundum, therefore demonstrating that the previous work only gave a superficial treatment of the mechanism of formation.

## Introduction

Flux methods are advantageous to crystal growth as it allows for the production of single crystals at significantly lower temperatures than melt growth methods.^1^ A molten oxide or combination of molten oxides, referred to as the flux, can act as a solvent and facilitate the dissolution of a solute, such as aluminum oxide, at a lower temperature than its melting point. The mechanism proceeds to promote crystal growth as a direct result of supersaturation, which can occur via one of three processes: evaporation of the flux, slow cooling of the solution, or by a thermal gradient.^1,2^ Single crystals of corundum (α-Al_2_O_3_), more specifically rubies (chromium-doped corundum), have been produced via various flux methods: PbO–B_2_O_3_, PbO–PbF_2_, PbO–PbF_2_–B_2_O_3_, AlF_3_–BaF_2_, PbF_2_–Bi_2_O_3_, and Na_3_AlF_6_.^3−5^ More recently, ruby crystals and other ruby-coated substrates have been produced via the isothermal evaporation of MoO_3_ flux.^6−10^

The synthesis of corundum via a MoO_3_ flux closely resembles catalytic Al_2_O_3_–MoO_3_ systems. Several observations made in Al_2_O_3_–MoO_3_ systems contradict the synthesis occurring via a conventional flux method. For example, a solid–solid interaction produces an intermediate phase of aluminum molybdate (Al_2_(MoO_4_)_3_), which is known to decompose into corundum above 900 °C, with uncertainty surrounding the phase transitions undergone by MoO_3_ and Al_2_(MoO_4_)_3_.^8,11^ For these reasons, in this work, we undertook the synthesis of corundum using a layered Al_2_O_3_–MoO_3_ system and directly observed the growth mechanism via optical imaging for the first time. This revealed a multistep process that is more complex than was previously thought. Based on the evidence presented in this work, the crystal growth process of corundum via MoO_3_ flux methods should be more accurately described as proceeding via a molten intermediate decomposition (MIND) mechanism.

## Experimental Section

### Sample Preparation

Identical syntheses were carried out in different receptacles: a 13 mL quartz vial and a 30 mL platinum crucible to assess the reaction across different temperature ranges. In each receptacle, 0.5 g of Al_2_O_3_ (99.7+ wt %, extra pure, Acros Organics) was placed at the bottom followed by a 2.1175 g MoO_3_ (99+ wt %, Acros Organics) layer on top. Each receptacle was then loosely covered with a platinum lid. The quartz vial was placed into a Carbolite HZS 12/900E three-zone tube furnace and taken from room temperature to 700 °C at 10 °C/min and then to 950 °C at 1 °C/min. The platinum crucible was placed into a Nabertherm L 9/11/SW Weighting Muffle furnace and taken from room temperature to 700 °C at 10 °C/min and then to 1100 °C at 1 °C/min. After a dwell time of 0 min, the furnaces were allowed to cool to room temperature before the receptacles were removed.

### Optical Imaging

A Nikon D3200 camera was trained on the quartz vial within a Carbolite HZS 12/900E three-zone tube furnace with an LED light source at the opposite end of the tube. A timelapse program was set on the camera to capture an image every 60 s throughout the experiment.

### Powder X-ray Diffraction

Powder X-ray diffraction (pXRD) measurements were carried out using a Bruker D8 advance diffractometer configured with Cu Kα radiation (λ = 1.54 Å). The patterns were collected at 0.011° step intervals over a 2θ range from 10 to 70° or 20 to 80°, both at 1 s per step.

### Scanning Electron Microscopy with Energy-Dispersive X-ray Analysis

Scanning electron microscopy (SEM) was carried out on a JEOL IT300 instrument. The samples were mounted onto aluminum stubs with carbon adhesive pads and sputter-coated with silver prior to imaging. Energy-dispersive X-ray analysis (EDXA) was achieved using an X-Max 80 mm^2^ detector and analyzed via the AZtec platform.

### Diffuse Reflectance UV–Vis Spectroscopy and CIE 1931 Standard Colorimetric Observer

Diffuse reflectance UV–vis spectroscopy was carried out on a PerkinElmer LAMBDA 650 UV/vis spectrophotometer using an integrating sphere between 360 and 830 nm with a step of 1 nm. The diffuse reflectance spectrum, β(λ), was used in combination with the CIE 1931 standard colorimetric observer to obtain the *XYZ* tristimulus values. The color-matching functions *x*(λ), *y*(λ), and *z*(λ) along with the spectral radiant power distribution for standard illuminant D_65_, *S*(λ), were taken from *Color Science: Concepts and Methods, Quantitative Data and Formulae*.^12^ The following formula was used to calculate *X*, *X* =  where *k* =  similarly, *Y* and *Z* were calculated.^12^ These were converted to CIE 1976 (L* a* b*)-space using the appropriate equations.

## Results and Discussion

**Figure 1:**
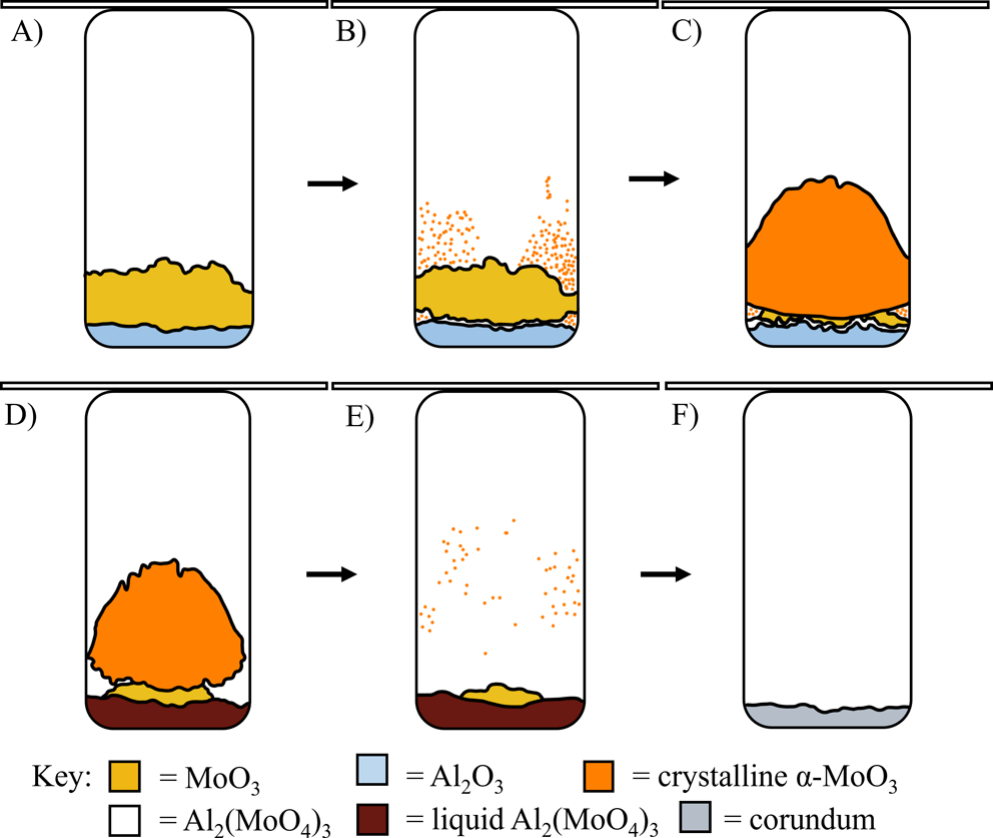
Schematic showing the molten intermediate decomposition (MIND) mechanism: (A) initial layered system, (B) formation of intermediate, (C) crystallization of α–MoO_3_ on the wall of vial, (D) start of melting, (E) end of melting, and (F) final product.

**Figure 2:**
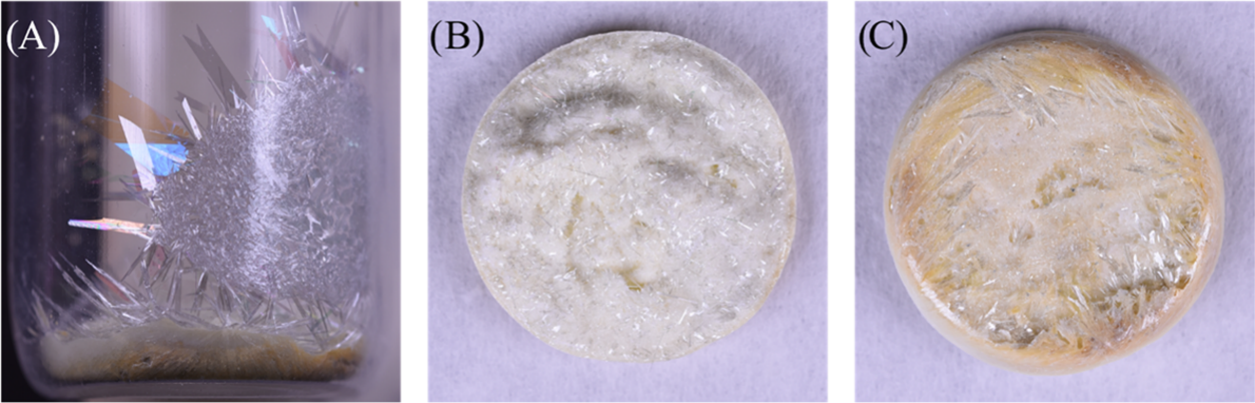
Post 950 °C product: (A) preremoval of the mixed solid disc and thin film-like crystals, (B) and (C) mixed solid disc, top and bottom, respectively.

The following crystal growth mechanism reflects the physical and chemical interactions witnessed in the layered Al_2_O_3_–MoO_3_ system between room temperature and 950 °C as captured by the timelapse images (see Video S1 in the Supporting information) and the result of heat treatment to 1100 °C. A schematic of the molten intermediate decomposition (MIND) mechanism is shown in Figure 1. The three key features of the mechanism are (1) the formation of an Al_2_(MoO_4_)_3_ layer via a solid–solid interaction between ∼705 and 860 °C (Figure 1B); (2) the melting of the Al_2_(MoO_4_)_3_ layer between approximately 870 and 890 °C (Figure 1D); and (3) the decomposition of Al_2_(MoO_4_)_3_ to corundum between 950 and 1100 °C (Figure 1F).

**Figure 3:**
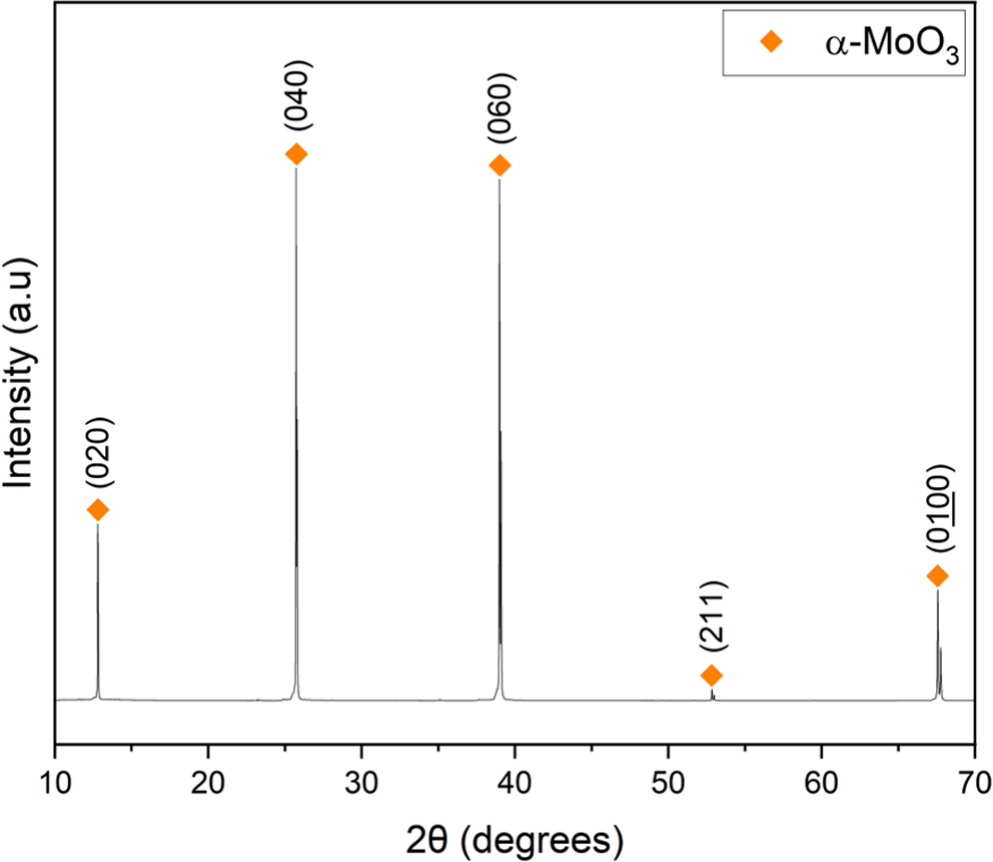
pXRD pattern of α–MoO_3_ thin film-like crystals removed from the quartz vial post 950 °C experiment.

**Figure 4:**
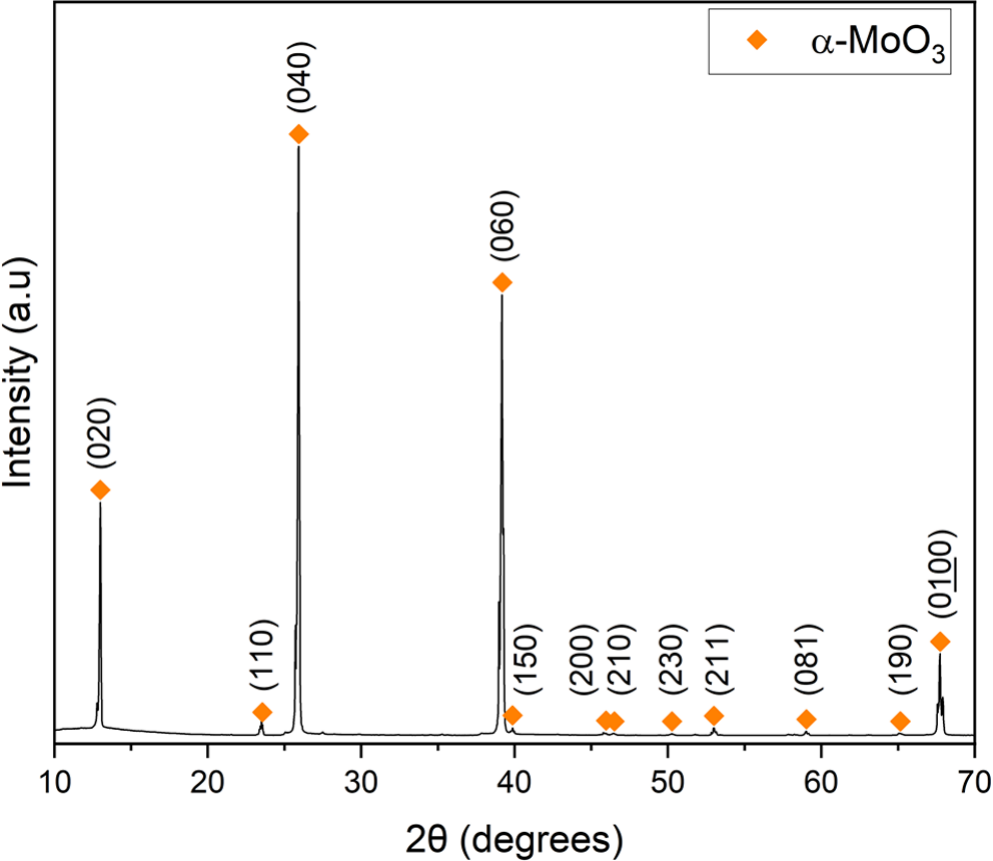
pXRD pattern of α–MoO_3_ thin film-like crystals removed from the walls of the quartz vial post 875 °C experiment.

**Figure 5:**
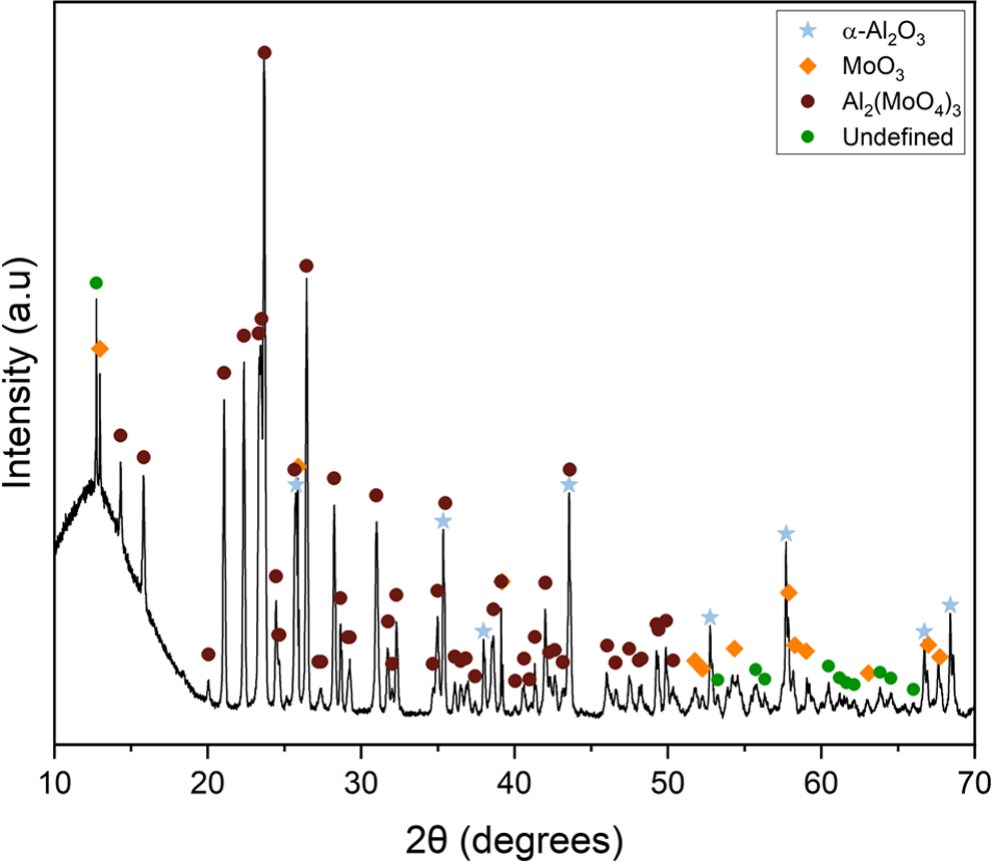
pXRD pattern of the intermediate layer from the quartz vial post 875 °C experiment.

In the temperature range of 20–700 °C, the MoO_3_ layer undergoes physical transformations. A color change from gray to yellow is seen upon heating as defects form within the crystal lattice.^13^ Also, the volume occupied by MoO_3_ decreases, which is likely due to sublimation (Figure 1A). At ∼709 °C, the formation of a crack appears below the interface between the layers (Figure 1B). The crack gradually extends over the course of 2 h 35 min as Al_2_(MoO_4_)_3_ is formed through a solid–solid interaction between Al_2_O_3_ and MoO_3_ (eq 1). It is thought that the MoO_3_ layer continuously sublimes throughout the mechanism into the atmosphere surrounding the system. As the underneath of the MoO_3_ layer becomes exposed, the atmosphere becomes saturated, and the deposition of α–MoO_3_ crystals can be seen on the walls of the vial (Figure 1B). The intermediate layer separates from the MoO_3_ layer above, and three distinct regions can be seen (Figure 1C). The deposition of α–MoO_3_ on the walls continues until ∼870 °C, and then it begins to melt along with the Al_2_(MoO_4_)_3_ layer (Figure 1D). Over the course of the following 20 min, liquid Al_2_(MoO_4_)_3_ flows down through the solid Al_2_O_3_ underneath and the MoO_3_ layer above sinks down into the liquid (Figure 1E). It is thought that the resulting mixture continues to form Al_2_(MoO_4_)_3_ through solid–liquid reactions and/or dissolution. This is followed by decomposition of Al_2_(MoO_4_)_3_ to corundum and gaseous MoO_3_ between 950 and 1100 °C (Figure 1F)
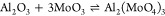
1A mixed solid disc with protrusions of thin film-like crystals remained at the bottom of the quartz vial after the 950 °C experiment. Also, thin film-like crystals were seen on the walls of the vial (Figure 2A). Images of the top and bottom of the disc can be seen in Figure 2B,C. The pXRD pattern for the thin film-like crystals correlates to α–MoO_3_ (JCPSD card 05-0508) preferentially orientated in the (0*k*0) plane where *k* = 2, 4, 6, or 10 (Figure 3). At approximately 870 °C, three distinct layers could be seen, and deposition of thin crystals on the walls of the vial was at a maximum. The intermediate layer and the thin crystals from the walls were obtained for characterization by repeating the experiment to 875 °C. The thin crystals were α–MoO_3_ (JCPSD card 05-0508) preferentially orientated in the (0*k*0) plane where *k* = 2, 4, 6, or 10 (Figure 4) and the intermediate layer had a dominant phase of Al_2_(MoO_4_)_3_ (JCPSD card 84-1652) (Figure 5). Additionally, sublimation of MoO_3_ from underneath the layer followed by growth of the intermediate layer was captured across a 30 min time frame (Figure S1). This suggests that Al_2_(MoO_4_)_3_ can be formed through gas–solid interactions between MoO_3_ and Al_2_O_3_.

**Figure 6:**
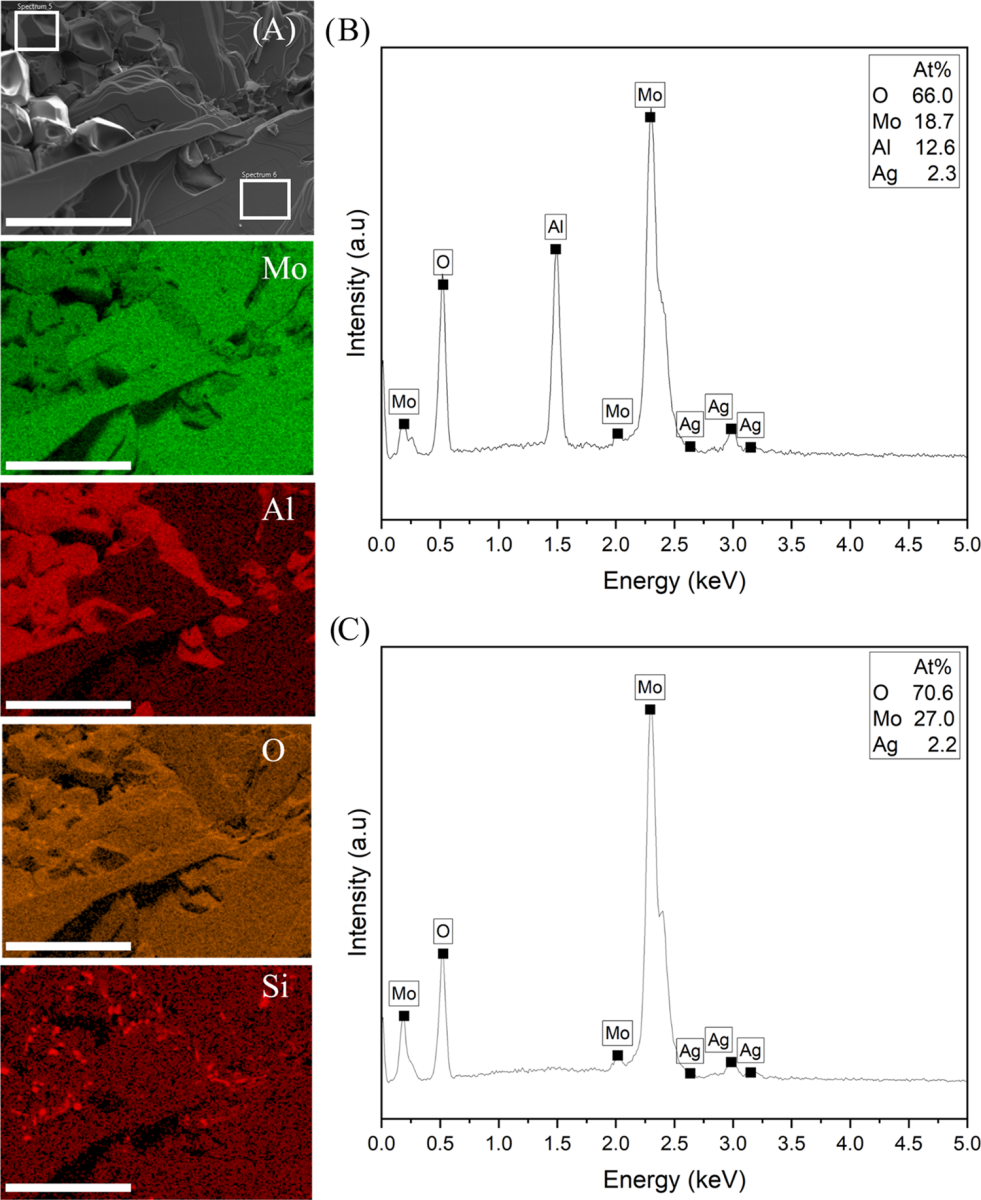
(A) SEM micrograph and EDXA maps from the top of the mixed solid disc highlighting two regions of different morphologies, (B) EDXA spectrum 5 associated with the blocky crystals, and (C) EDXA spectrum 6 associated with the thin film-like crystals. Scale bar = 100 μm.

**Figure 7:**
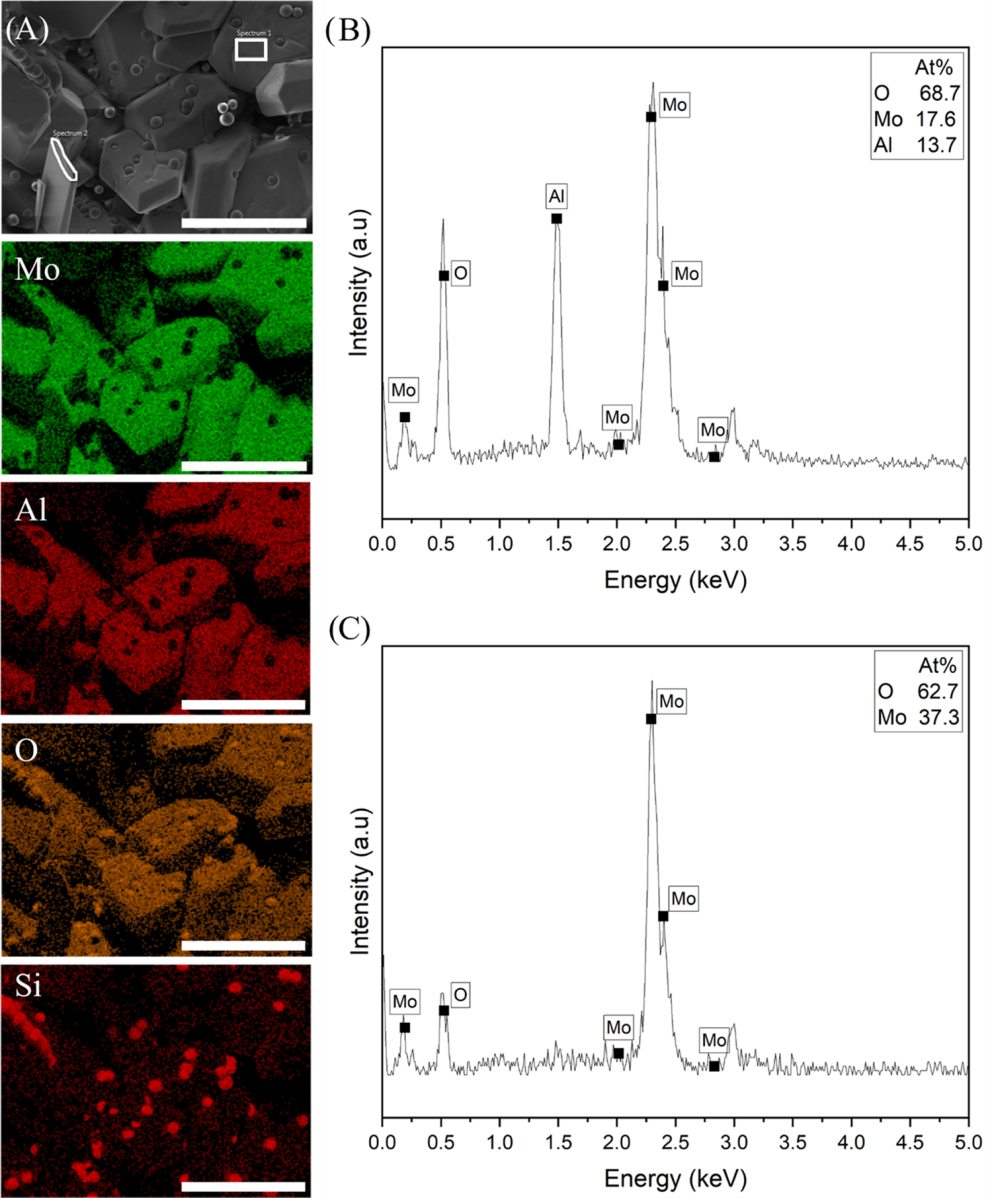
(A) SEM micrograph and EDXA maps from the bottom of the mixed solid disc highlighting two regions of different morphologies, (B) EDXA spectrum 1 associated with the blocky crystals, and (C) EDXA spectrum 2 associated with the thin film-like crystals. Scale bar = 100 μm.

**Figure 8:**
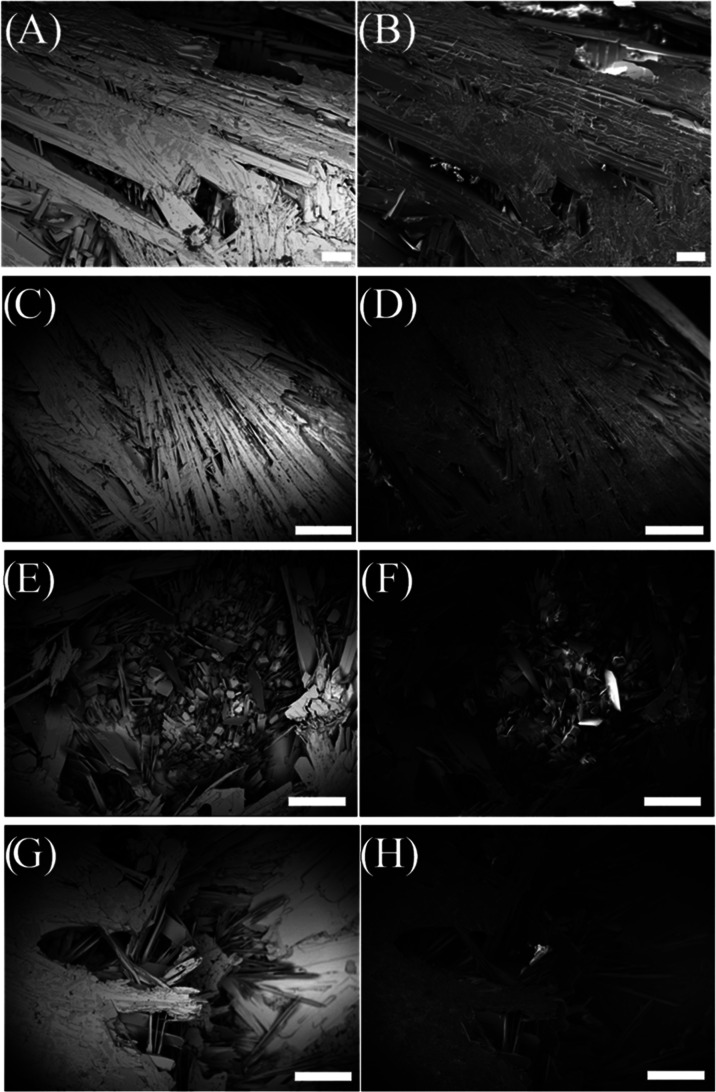
SEM micrographs from the bottom of the mixed solid disc; panels (A, C, E, and G) from backscattered electron detection and panels (B, D, F, and H) from secondary electron detection in the same areas, respectively. Scale bars = 500 μm, except for panels (A) and (B) where scale bars = 100 μm.

SEM and EDXA of the mixed solid disc reveal heterogeneous regions of Al/Mo/O or Mo/O. Elemental mapping from the middle of the top and bottom of the disc is shown in Figures 6 and 7, respectively. Three different morphologies can be seen in the micrographs: an aggregation of blocky crystals of between 8 and 80 μm with depressions similar to hopper crystals, thin film-like/lamellar structures, and spherical particles. Based on the elemental mapping, the spherical particles are presumed to be silicon dioxide originating from the quartz vial. These spherical particles are not present when a platinum crucible is used. EDXA spectra for each morphology on the top and bottom of the disc can be seen in Figures 6 and 7, respectively. The spectra in Figure 6B show the composition of the blocky crystals to be Al_2.00_Mo_3.00_O_10.44_, and the spectra in Figure 6C show the composition of the thin film-like crystals to be MoO_2.61_. The spectra in Figure 7B show the composition of the blocky crystals to be Al_2.00_Mo_2.56_O_10.00_, and the spectra in Figure 7C show the composition of the thin film-like crystals to be MoO_1.68_. The compounds under the disc were more oxygen-deficient compared to those at the top of the disc. This is confirmed further by the backscattered electron micrographs from the bottom of the disc of regions containing the thin film-like crystals, as various shades of gray can be seen in correlation to different atomic weights (Figure 8). As stoichiometric ratios of Al_2_(MoO_4_)_3_ or MoO_3_ were not detected in the analyzed areas, this suggests that after the intermediate Al_2_(MoO_4_)_3_ layer has melted, there is a degree of mixing and/or dissolution with the remaining solid Al_2_O_3_ and MoO_3_.

The lack of Al_2_O_3_ in the mixed solid base and the formation of thin film-like crystals of α–MoO_3_, provided sufficient oxygen, is consistent with the high chemical adsorption of molybdenum oxides onto the surface of Al_2_O_3_. It has been shown previously that at low surface concentrations, molybdenum oxides undergo chemical adsorption as [MoO_4_] polyhedrons at tetrahedral sites and/or [MoO_6_] at octahedral sites depending on whether the dispersion threshold has been reached.^14^ As higher surface concentrations are reached, crystalline orthorhombic MoO_3_ will form as [MoO_6_] layers parallel to the [010] plane.^14^ The adsorption of [MoO_4_] polyhedrons on the surface of Al_2_O_3_ is therefore promoting the formation of the Al_2_(MoO_4_)_3_ intermediate layer.^15^

**Figure 9:**
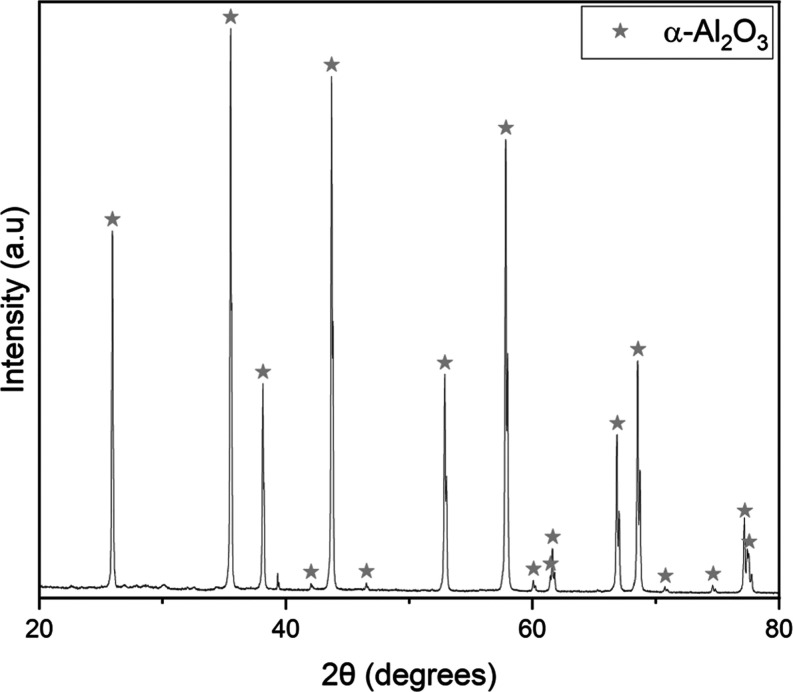
pXRD pattern of the product from the platinum crucible after a 1100 °C experiment.

The product collected from the platinum crucible, post 1100 °C experiment, was characterized as corundum via pXRD (JCPSD card 46-1212) (Figure 9). The corundum was light bluish-gray in color and can be defined in CIE (L* a* b*) color space as L* = 76.65, a* = −1.09, and b* = −6.20. The coloration of the corundum is likely due to Mo impurities. Similar gray–blue Al_2_O_3_–MoO_3_ systems have been reported along with their performance as pigments,^14^ so the corundum synthesized in this work could potentially find use as a nonhazardous inorganic pigment.

## Conclusions

A new crystal growth process for the synthesis of corundum, the molten intermediate decomposition (MIND) mechanism, was discovered through the direct observation of an Al_2_O_3_–MoO_3_ system. The three key features of this mechanism are (1) the formation of an Al_2_(MoO_4_)_3_ intermediate layer through a solid–solid interaction in the temperature range of ∼705–860 °C; (2) the melting of the Al_2_(MoO_4_)_3_ layer between approximately 870 and 890 °C; and (3) the decomposition of Al_2_(MoO_4_)_3_ to corundum between 950 and 1100 °C. This work gives a deeper understanding of the crystal growth processes involved in the synthesis of corundum from Al_2_O_3_–MoO_3_, which, until now, were presumed to occur via the conventional flux method.

## Supporting Information Available

Timelapse video clip of the 950 °C experiment (Video S1) (AVI)
Images from an 875 °C experiment displaying the sublimation of MoO_3_ followed by the growth of Al_2_(MoO_4_)_3_ (Figure S1) (PDF)

## Supplementary Material

ao3c07772_si_001.avi

## References

[ref1] Elwell D.; Scheel H. J. Crystal Growth from High-Temperature Solutions; Academic Press Inc. Ltd, 1975.

[ref2] Tachibana M. Beginner’s Guide to Flux Crystal Growth; Springer Japan: Tokyo, 2017 10.1007/978-4-431-56587-1.

[ref3] Nelson D. F.; Remeika J. P. Laser Action in a Flux-Grown Ruby. J. Appl. Phys. 1964, 35 (3), 522–529. 10.1063/1.1713406.

[ref4] Linares R. C. Properties and Growth of Flux Ruby. J. Phys. Chem. Solids 1965, 26 (12), 1817–1818. 10.1016/0022-3697(65)90214-3.

[ref5] Watanabe K.; Sumiyoshi Y.; Sunagawa I. Growth Mechanism of Corundum Crystals from Cryolite (Na_3_AlF_6_) Flux. J. Cryst. Growth 1977, 42 (C), 293–298. 10.1016/0022-0248(77)90209-3.

[ref6] Oishi S.; Teshima K.; Kondo H. Flux Growth of Hexagonal Bipyramidal Ruby Crystals. J. Am. Chem. Soc. 2004, 126 (15), 4768–4769. 10.1021/ja049678v. 15080667

[ref7] Teshima K.; Takano A.; Suzuki T.; Oishi S. Unique Coating of Ruby Crystals on an Aluminum Oxide Wall by Flux Evaporation. Chem. Lett. 2005, 34 (12), 1620–1621. 10.1246/cl.2005.1620.

[ref8] Teshima K.; Matsumoto K. I.; Kondo H.; Suzuki T.; Oishi S. Highly Crystalline Ruby Coating on α-Al_2_O_3_ Surfaces by Flux Evaporation. J. Ceram. Soc. Jpn. 2007, 115 (1342), 379–382. 10.2109/jcersj.115.379.

[ref9] Ayuzawa S.; Suzuki S.; Hidaka M.; Oishi S.; Teshima K. Epitaxial Growth of Ruby Crystal Films on Sapphire Crystal Substrates and Solubility of Aluminum Oxide in Molybdenum Trioxide Flux. Cryst. Growth Des. 2019, 19 (7), 4095–4100. 10.1021/acs.cgd.9b00483.

[ref10] Ayuzawa S.; Suzuki S.; Hidaka M.; Oishi S.; Teshima K. Effect of Holding Temperature on Growth of Ruby Crystal Films via Molybdenum Trioxide Flux Evaporation-Solubility of Aluminum Oxide, Growth Rate, and Material Balance. Cryst. Growth Des. 2020, 20 (3), 2019–2026. 10.1021/acs.cgd.9b01674.

[ref11] Ibrahim A. A.; El-Shobaky G. A. Solid-Solid Interactions in the MoO_3_-Al_2_O_3_, System. Thermochim. Acta 1989, 147 (1), 175–188. 10.1016/0040-6031(89)85173-1.

[ref12] Wyszecki G.; Stiles W. S. Color Science: Concepts and Methods, Quantitative Data and Formulae, 2nd ed.; John Wiley & Sons, Inc: Nashville, TN, 1982.

[ref13] Silvestri S.; Kubaski E. T.; Sequinel T.; Pianaro S. A.; Varela J. A.; Tebcherani S. M. Optical Properties of the MoO_3_-TiO_2_ Particulate System and Its Use as a Ceramic Pigment. Part. Sci. Technol. 2013, 31 (5), 466–473. 10.1080/02726351.2013.773388.

[ref14] Dondi M.; Matteucci F.; Baldi G.; Barzanti A.; Cruciani G.; Zama I.; Bianchi C. L. Gray–Blue Al_2_O_3_–MoO_x_ Ceramic Pigments: Crystal Structure, Colouring Mechanism and Performance. Dyes Pigm. 2008, 76 (1), 179–186. 10.1016/j.dyepig.2006.08.021.

[ref15] Spevack P. A.; McIntyre N. S. A Raman and XPS Investigation of Supported Molybdenum Oxide Thin Films. 1. Calcination and Reduction Studies. J. Phys. Chem. A 1993, 97 (42), 11020–11030. 10.1021/j100144a020.

