# Supplementary material for: New and Unforeseen Crystal Growth Processes for a Metal Oxide
Source: ACS Omega. 2023 Dec 12;8(51):49327–33. doi: 10.1021/acsomega.3c07772 (PMC10753548; doi:10.1021/acsomega.3c07772)
Supplement: Supplementary file 2 — ao3c07772_si_002.pdf [file ao3c07772_si_002.pdf]

Supporting information for New and Unforeseen Crystal Growth Processes for a Metal Oxide

*Michaela E. Whitehurst and Simon R. Hall **

School of Chemistry, University of Bristol, Cantock's Close, Bristol BS8 1TS, United Kingdom

Video S1. Timelapse video clip of the 950 °C experiment. (Available online)

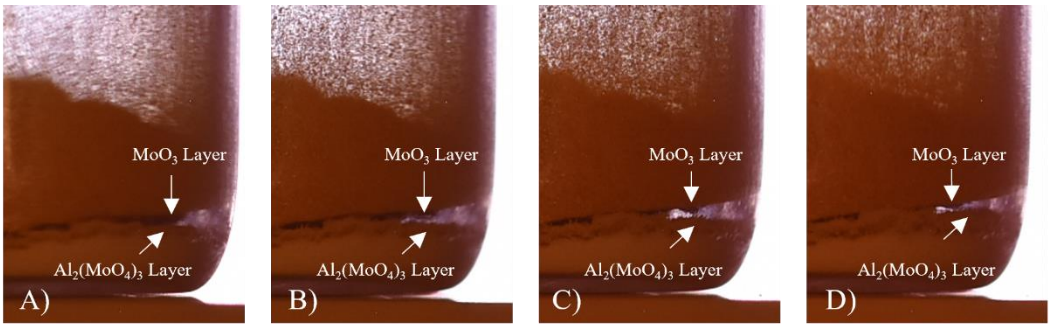

Figure S1. Images from an 875 °C experiment displaying the sublimation of MoO_3 followed by the growth of $Al_2(MoO_4)_3$.
